# Supplementation with Selenium and Coenzyme Q10 Reduces Cardiovascular Mortality in Elderly with Low Selenium Status. A Secondary Analysis of a Randomised Clinical Trial

**DOI:** 10.1371/journal.pone.0157541

**Published:** 2016-07-01

**Authors:** Urban Alehagen, Jan Alexander, Jan Aaseth

**Affiliations:** 1 Division of Cardiovascular Medicine, Department of Medical and Health Sciences, Linköping University, Linköping, Sweden; 2 Norwegian Institute of Public Health, Oslo, and Norwegian University of Life Sciences (NMBU), Ås, Norway; 3 Research Department, Innlandet Hospital Trust, Elverum, Norway; 4 Hedmark University College, Elverum, Norway; Indiana University Richard M. Fairbanks School of Public Health, UNITED STATES

## Abstract

**Background:**

Selenium is needed by all living cells in order to ensure the optimal function of several enzyme systems. However, the selenium content in the soil in Europe is generally low. Previous reports indicate that a dietary supplement of selenium could reduce cardiovascular disease but mainly in populations in low selenium areas. The objective of this secondary analysis of a previous randomised double-blind placebo-controlled trial from our group was to determine whether the effects on cardiovascular mortality of supplementation with a fixed dose of selenium and coenzyme Q10 combined during a four-year intervention were dependent on the basal level of selenium.

**Methods:**

In 668 healthy elderly individuals from a municipality in Sweden, serum selenium concentration was measured. Of these, 219 individuals received daily supplementation with selenium (200 μg Se as selenized yeast) and coenzyme Q10 (200 mg) combined for four years. The remaining participants (n = 449) received either placebo (n = 222) or no treatment (n = 227). All cardiovascular mortality was registered. No participant was lost during a median follow-up of 5.2 years. Based on death certificates and autopsy results, all mortality was registered.

**Findings:**

The mean serum selenium concentration among participants at baseline was low, 67.1 μg/L. Based on the distribution of selenium concentration at baseline, the supplemented group was divided into three groups; <65 μg/L, 65–85 μg/L, and >85 μg/L (45 and 90 percentiles) and the remaining participants were distributed accordingly. Among the non-treated participants, lower cardiovascular mortality was found in the high selenium group as compared with the low selenium group (13.0% vs. 24.1%; *P* = 0.04). In the group with the lowest selenium basal concentration, those receiving placebo or no supplementation had a mortality of 24.1%, while mortality was 12.1% in the group receiving the active substance, which was an absolute risk reduction of 12%. In the middle selenium concentration group a mortality of 14.0% in the non-treated group, and 6.0% in the actively treated group could be demonstrated; thus, there was an absolute risk reduction of 8.0%. In the group with a serum concentration of >85 μg/L, a cardiovascular mortality of 17.5% in the non-treated group, and 13.0% in the actively treated group was observed. No significant risk reduction by supplementation could thus be found in this group.

**Conclusions:**

In this evaluation of healthy elderly Swedish municipality members, two important results could be reported. Firstly, a low mean serum selenium concentration, 67 μg/L, was found among the participants, and the cardiovascular mortality was higher in the subgroup with the lower selenium concentrations <65 μg/L in comparison with those having a selenium concentration >85 μg/L. Secondly, supplementation was cardio-protective in those with a low selenium concentration, ≤85 at inclusion. In those with serum selenium>85 μg/L and no apparent deficiency, there was no effect of supplementation. This is a small study, but it presents interesting data, and more research on the impact of lower selenium intake than recommended is therefore warranted.

**Trial Registration:**

Clinicaltrials.gov NCT01443780

## Introduction

Selenium is important for many cellular functions in the body. In anti-oxidative defence several selenoproteins, including glutathione peroxidases (GPX) and thioredoxin reductase, are important. Selenoprotein P (SEPP1), which is the most abundant selenoprotein in the blood, and plays an important role in supplying other tissues with selenium, also has anti-oxidative properties. The human selenoproteome has 25 genes encoding selenoproteins, of which all contain the amino acid selenocysteine [1–3]. In plasma, SEPP1 constitutes about 60% and GPX constitutes about 25% of the selenium-containing proteins [4]. To obtain an optimal function of SEPP1, an intake of approximately105 μg/day has been found to be necessary [5].

As the selenium content of the soil varies in different parts of the world, the estimated selenium intake varies between populations from different geographical regions. It has been estimated that the selenium intake in a US population is about 120 μg/day [6, 7], whereas lower intakes, even below 50 μg/day in some cases, have been reported from different European countries, including Sweden [1, 8–12].

Until now, most of the discussions regarding optimal selenium intake have been based on the selenium intake required in order to obtain the optimal function of the enzyme GPX in blood or plasma. However, recent data indicates that a more appropriate indicator of optimal selenium intake might be the level required to obtain the optimal expression of SEPP1 [13].

Studies on cardiovascular mortality in population groups given dietary selenium supplementation have shown conflicting results. However, background dietary selenium intake varies widely. The most obvious reason for the discrepancies between studies is that in order to obtain positive effects of nutrient supplementation regardless of type, a documented deficiency or suboptimal supply of the substance to be supplemented should exist in the population under investigation.

As the calculated selenium intake in the general population in the US is above 100 μg/day, full expression of SEPP1 and most other selenoproteins is expected; hence, it is not surprising that investigations such as the US-based SELECT and the NPC trials could not demonstrate a reduction in cardiovascular mortality through dietary supplementation with selenomethioneine or selenized yeast, respectively [14].

However, there are observational studies showing lower selenium levels in patients with myocardial infarction than in controls, and increased risk for myocardial infarction could be calculated in those with the lowest selenium status [15]. An early report by Salonen and co-workers found an increased risk of cardiovascular mortality with a selenium intake of less than 45 μg/day [16], and Blankenberg et al. were able to demonstrate an increased risk for those with a low selenium intake, as evaluated from determinations of GPX 1 activity in blood [17]. In a rat model, in comparison with rats on a low selenium diet, those supplemented with selenium had a reduced infarct size and less remodelling [18].

Our Nordic group recently reported data on cardiovascular mortality obtained from an elderly healthy population, where the median serum selenium concentration was 67 μg/L, and where those in the lowest quartile had a mean selenium concentration of 57 μg/L [19]. The latter group had a substantially increased risk of cardiovascular mortality in comparison with the second to the fourth quartiles, even when adjusted for several clinical variables known to influence cardiovascular risk, such as male gender, smoking, ischemic heart disease, chronic obstructive pulmonary disease, and reduced cardiac function (ejection fraction (EF)<40%) [19].

The aim of this secondary analysis of a previously published main study [20] was to determine whether there were different cardiovascular mortality risks for three different strata of selenium concentrations in serum, and whether the possible impact of selenium supplementation on cardiovascular mortality differed depending on the baseline serum level of selenium in the same strata and applying the same follow-up period.

## Materials and Methods

### Study population

This is a secondary post-hoc analysis of a recently published study where the participants in the study were recruited from a rural municipality of 10,300 inhabitants in the south-east of Sweden [19].

All citizens in the municipality aged between 70–80 years were invited to participate in the study. From a population of 1,130 individuals living in the municipality of the chosen age-range, 675 consented to participate. Of these, 668 agreed that blood samples could be drawn, and they were thus included in the present study for analysis of selenium. All cardiovascular mortality was analysed in this study population.

**Fig 1:**
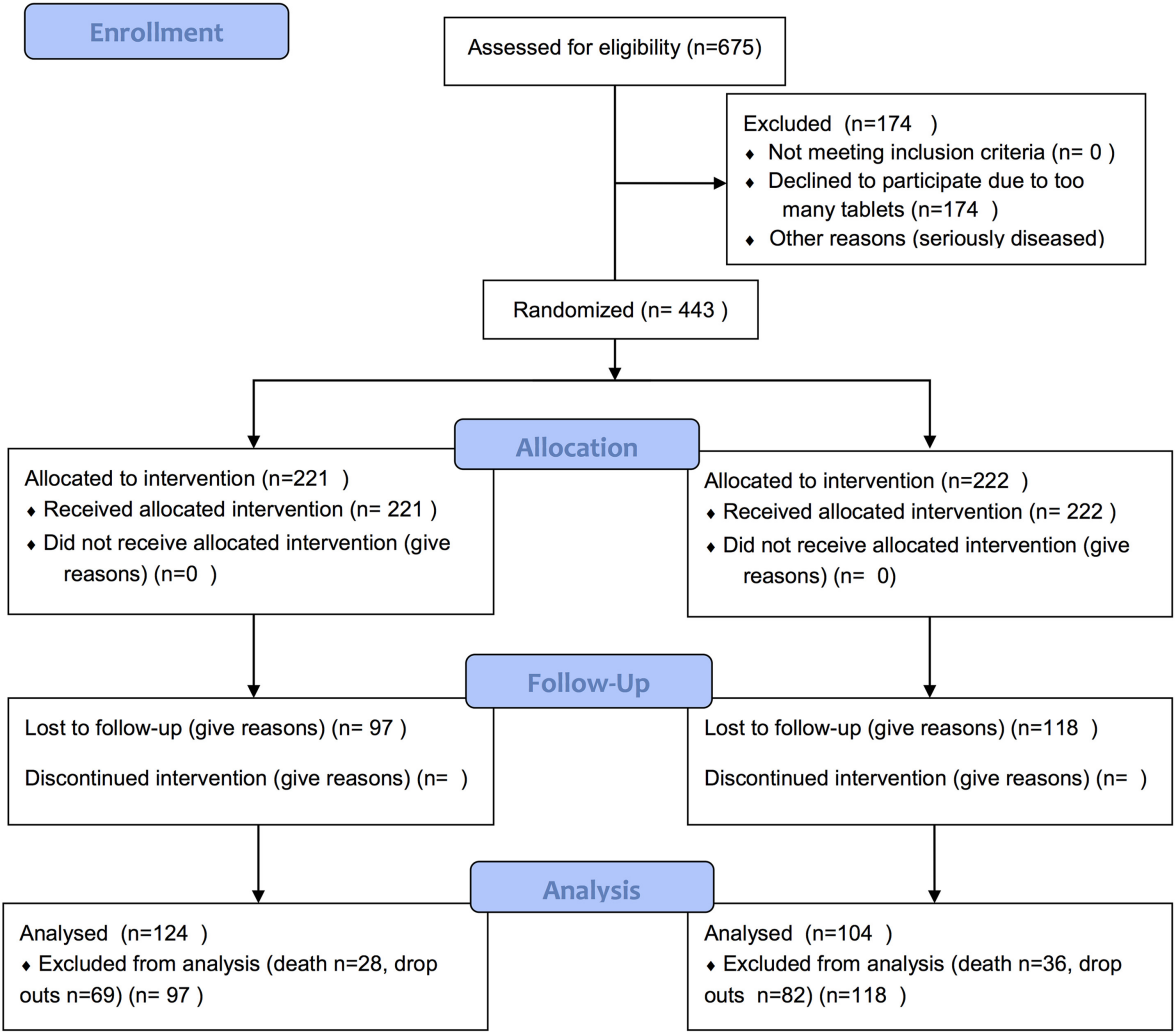
CONSORT flow chart of the study.

Of the 668 included, a total of 443 agreed to participate in an intervention study. In the intervention project, which was published earlier [20], 219 persons were given the active supplement of selenium + coenzyme Q10 (active treatment group), and 222 persons received the placebo supplement (placebo group), Fig 1.

The first participant was included in January 2003, and the last participant concluded the study in February 2010. All participants were examined by one of three experienced cardiologists. In each case, a new clinical history was recorded, a clinical examination was performed, Doppler-echocardiography and a new electrocardiogram were carried out, and the New York Heart Association (NYHA) functional class was assessed. The latter grades how a patient with heart disease experiences symptoms of tiredness, breathlessness or chest pain; it is graded from I-IV, where IV represents symptoms at rest. Blood pressure was measured in the right arm with the participant resting in a supine position.

All participants were supplemented for 48 months, and were re-examined at the end of each six-month period. The median follow-up time was 5.2 years. All cardiovascular mortalities were registered. The mortality information was obtained from the National Board of Health and Welfare in Sweden, which registers all deaths of Swedish citizens.

The study population for the current risk evaluation consisted of 668 individuals, which included 449 individuals serving as controls, who received placebo treatment (n = 222) and 227 individuals who did not receive any treatment, in addition to 219 participants given active supplementation. To increase the statistical power we used a combined control group consisting of those who received the placebos and the individuals who did not receive any treatment. To check whether this was an appropriate procedure, analyses were also carried out using the placebos alone as a control group. Serum selenium analyses were performed in samples collected from all participants at inclusion and from 98 randomised participants at 48 months.

The study was approved by the Regional Ethical Committee (Diary no. 03–176) and conforms to the ethical guidelines of the 1975 Declaration of Helsinki. Written, informed consent was obtained from all patients.

The study protocol has previously been published [21]. The intervention study was registered at Clinicaltrials.gov, and has the identifier NCT01443780.

### Supplementation

All participants that took part in the intervention study (n = 443) were randomised in blocks of six in a double-blind manner and given either a combination of 200 mg/day of coenzyme Q10 capsules (Bio-Quinon 100 mg twice daily, Pharma Nord, Vejle, Denmark) and 200 μg selenium/day of organic selenium yeast tablets (SelenoPrecise 200 μg, Pharma Nord, Vejle, Denmark), or a placebo. The study supplementation was taken in addition to regular medication. Study medication that was not consumed (active drug and placebo) was returned and counted. Due to the fact that two of the participants did not deliver blood samples for selenium analyses the study population in this evaluation consisted of 441individuals.

The selenium source was patented selenium yeast, SelenoPrecise^®^, of a pharmaceutical quality and documented batch-to-batch stability in its composition of selenium species [22–24]. Previous results from the Precise pilot studies showed low levels of adverse effects and good absorption [23] in doses up to 300 μg/day. SelenoPrecise^®^ has been approved as a pharmaceutical drug in Denmark by the Danish Medicines Agency for many years (appr. No. 6233603).

The coenzyme Q10 preparations have shown good absorption and efficacy in previous controlled trials [25, 26], and the capsules were identical to medicinal quality capsules registered for heart failure in a European Union Member State (Myoqinon^®^, authorization no. OGYI 11494–2010).

### Blood samples

The blood samples were collected while the participants were resting and in a supine position. Pre-chilled EDTA vials were used. The vials were centrifuged at 3000 *g*, +4°C, plasma was transferred to new vials, and was frozen at -70°C. No samples were thawed until the analyses were to be carried out.

### Selenium analysis

The serum selenium analyses were performed using ICP-MS methodology at an Agilent 700 platform at Kompetenzzentrum für komplementärmedizinische Diagnostik, Zweigniederlassung der synlab MVZ Leinfelden GmbH (Leinfelden-Echterdingen, Germany). The accuracy of the measurements was checked by analysing two external reference materials with certified values of 63 μg/L and 103 μg/L (control programme offered by the Society for Advancement of Quality Assurance in Medical Laboratories, INSTAND e.V., Düsseldorf, Germany), showing values within 90–110% of certified concentrations. A round-robin test with INSTAND e.V. was passed adequately. The precision of the method, checked by repetitive analyses of the same sera, showed an average coefficient of variation of 5.7%.

### Echocardiography

Doppler echocardiographic examinations (Accuson *XP-128c*) were performed with the participant in the left lateral position. The cardiac function readings (expressed as EF) were categorized into four classes, with interclass limits placed at 30%, 40% and 50%.[27, 28] Normal systolic function was defined as EF≥ 50%, while severely impaired systolic function was defined as EF< 30%.

### Statistical methods

The descriptive data are presented as percentages or mean ± SD. The student’s unpaired two-sided T-test was used for continuous variables. For variables on the nominal scale level, multiple Chi square tests were performed. Kaplan-Meier analyses illustrating the association between levels of selenium and cardiovascular mortality and the effect of intervention during a follow-up of 2,500 days are presented. The basal level of selenium was analysed in 668 participants, whereas when evaluating the effect of the intervention, those on active treatment (n = 219) were compared against those not on treatment. Censored participants were those still living at the end of the study, or those who had died for reasons other than cardiovascular diseases. Completed participants were those who had died due to cardiovascular diseases.

The participants receiving active treatment with selenium and Q10 (n = 219) were divided into groups according to the distribution of serum selenium at baseline as follows: those above the 10 percentile (n = 20) had a value >85 ug/L and the rest (n = 199) were divided in two equal groups: those with s-Se 65–85 ug/L (n = 100) (45–90 percentile) and those with a s-Se <65 ug/L (n = 99) (<45 percentile). Participants who did not receive any active treatment (n = 449) were classified accordingly: >85 ug/L, n = 57; 65–85 ug/l, n = 193; < 65 ug/L, n = 199. Of those, the participants receiving a placebo (n = 221) were distributed as follows; <65 ug/L n = 101, 65–85 ug/L n = 93, and finally >85 ug/L n = 27. The participants receiving a placebo and the controls were compared regarding cardiovascular mortality without finding any significant difference, which is why it was possible to amalgamate the two groups into one in the following evaluations.

Assessment of the risk of cardiovascular mortality for the three strata in the serum selenium was performed using univariate and multivariate Cox proportional hazard regressions. Factors that influence measured selenium levels include certain diseases and smoking. In the multivariate model, adjustments were made for the following clinical variables: male gender, smoking, ischemic heart disease, and EF <40% according to echocardiography.

P-values <0.05 were considered significant, based on two-sided evaluations. All data were analysed using Statistica v12.0 software (Statsoft Inc, Tulsa, OK, USA).

## Results

### Population baseline characteristics

The study population was divided into three groups, the lowest having a serum selenium concentration of <65 μg/L, the medium having a concentration between 65–85 μg/L, and the highest having a concentration of >85 μg/L. The characteristics of these groups are presented in Table 1.

**Table 1:** Baseline characteristics of the study population divided into a serum selenium concentration of <65 μg/L, 65–85 μg/L or a selenium concentration >85 μg/L.

*Variable*	Selenium concentration< 65 μg/L	Selenium concentration 65–85 μg/L	Selenium concentration >85 μg/L	*P*-value
n	298	293	77	
Age, mean	78	77	78	0.46
*Males*/Females, n	147/150	143/150	28/49	0.11
Smokers, n (%)	33 (11.1)	21 (7.2)	9 (11.7)	0.21
Diabetes, n (%)	63 (21.1)	69 (23.5)	14 (18.2)	0.55
Hypertension, n (%)	222 (74.5)	220 (75.1)	58 (75.3)	0.98
COPD, n (%)	35 (11.7)	35 (11.9)	10 (13.0)	0.96
IHD, n (%)	90 (30.2)	60 (20.5)	11 (14.3)	χ^2^:12.22; *P* = 0.002
NYHA I, n (%)	131 (44.0)	146 (49.8)	40 (51.9)	0.25
NYHA II, n (%)	94 (31.5)	87 (29.7)	23 (30.0)	0.88
NYHA III, n (%)	62 (20.8)	56 (19.1)	13 (16.9)	0.71
NYHA IV, n (%)	4 (1.3)	0	0	
Unclassified, n (%)	7 (2.3)	4 (1.4)	1 (1.3)	NS
ACEI, n (%)	64 (21.5)	53 (18.1)	20 (26.0)	0.27
ARB, n (%)	10 (3.4)	16 (5.5)	4 (5.2)	0.44
Digitalis, n (%)	17 (5.7)	13 (4.4)	3 (3.9)	0.70
Diuretics, n (%)	102 (34.2)	103 (35.2)	26 (33.8)	0.96
EF<40%, n (%)	29 (9.7)	25 (8.5)	6 (7.8)	0.81
Hb<120g/L, n (%)	44 (14.8)	27 (9.2)	9 (11.7)	0.12

Note: ACEI; ACE- inhibitors; ARB: Angiotensin receptor blocker; COPD: Chronic obstructive pulmonary disease; EF: Ejection fraction. EF as measured by echocardiography; IHD: Ischemic heart disease; NS: Non-significant difference; NYHA: New York Heart Association functional class

A smaller proportion of males in relation to females could be seen in the group with the higher selenium concentration (Table 1). In the lowest group, significantly more participants had a history of ischemic heart disease as compared to those in the higher concentration groups (30.2% vs. 20.5% and 14.3% respectively; χ^2^:12.22; *P* = 0.002). Evaluating the functional capacity, a few participants, four out of 298 (1.3%), in the lowest group were classified as having symptoms already at rest (NYHA class IV), whereas none in the groups with the higher selenium concentration were classified as having that degree of impaired functional capacity. No other significant differences could be registered between the two groups at baseline.

The mean follow-up period of the total study population (n = 668) was 1870 days (SD 499 days).

### Serum selenium concentrations and cardiovascular mortality

In the total study population, the mean serum selenium concentration was 67.1 μg/L (SD 16.8), and of the total study population, 85.5% (588 out of 688) had a selenium concentration of <85 μg/L.

The cardiovascular mortality in those without any selenium supplementation and with a mean selenium concentration of <65 μg/L (n = 199) was compared to the group with a selenium concentration of >85 μg/L (n = 57). There was a significant difference between the two groups (*P* = 0.03) with a cardiovascular mortality of 28.1% (56 out of 199) in the low concentration group, and a cardiovascular mortality of 14.0% (eight out of 57) in the high concentration group. As the size of the high concentration group was small, the results should be interpreted with caution. The cardiovascular mortality in the group with a serum selenium concentration of 65–85 μg/L was 11.6% (24 out of 206).

### Effect of intervention in different selenium concentration groups

**Fig 2:**
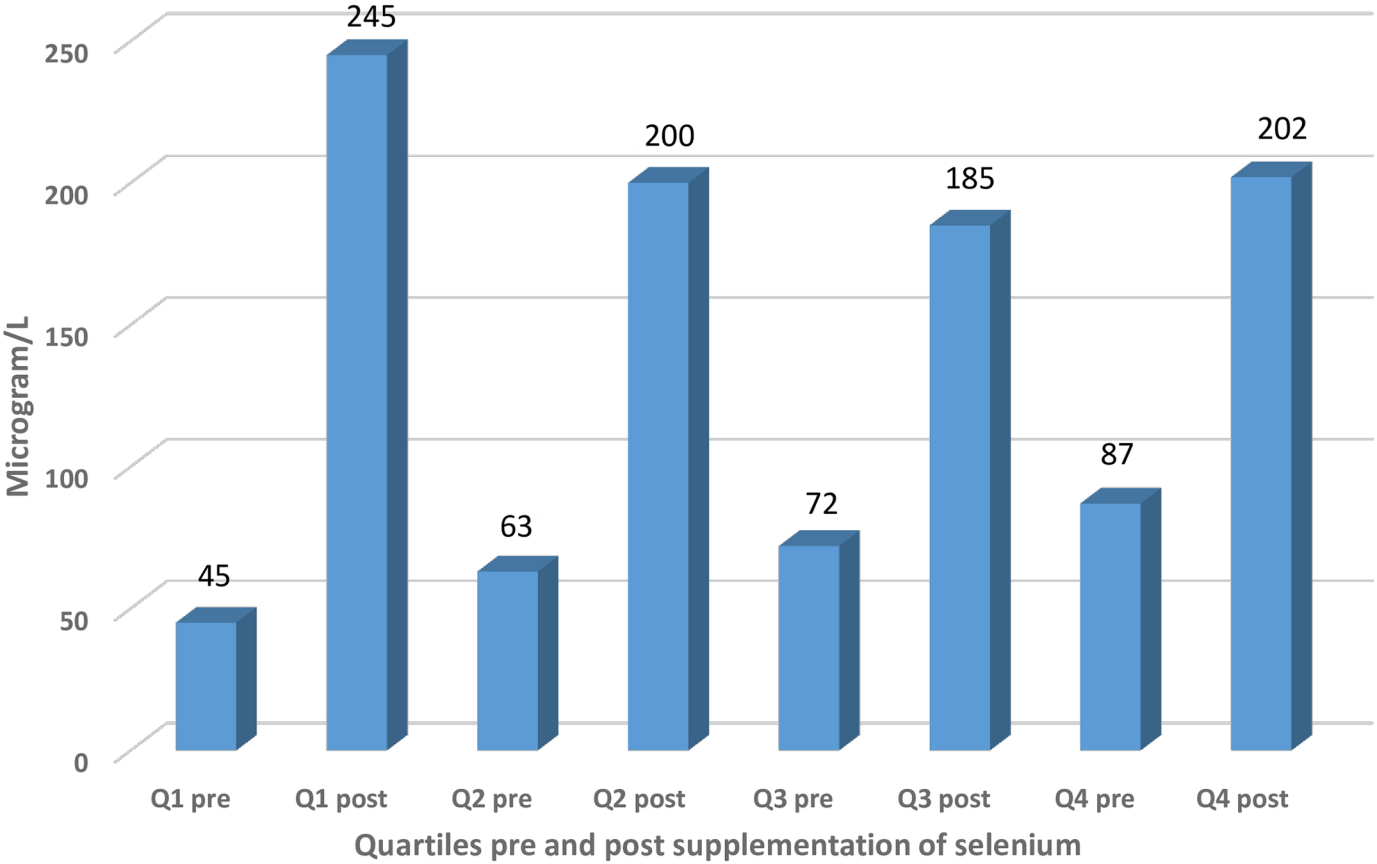
Histogram illustrating the mean concentration of serum selenium divided into the four quartiles based on the basal measurements, and effect of supplementation with selenium.

We evaluated the effect of the 48-month intervention with selenium and coenzyme Q10 versus placebo, as described above. In 441 of the participants who took part in the intervention, 219 received active substances, and 222 received a placebo. During the follow-up period 86 participants died, and 129 (29.1%) decided not to continue the intervention, mainly because they had to take too many tablets/capsules each day [20]. The evaluation was based on the three groups with different basal levels of serum selenium concentration as described above: <65 μg/L (representing those with a low selenium concentration), those with a concentration of 65–85 μg/L (representing a middle group), and those with >85 μg/L (representing those with a high concentration at inclusion). After the 48-month intervention, measurements were performed on 98 individuals of the 668 that constituted the total study population. In those supplemented with 200 ug Se as selenized yeast, serum levels after 48 months were similar (from 185 to 245 ug/L), irrespective of the quartile at baseline, whereas those receiving a placebo remained at about the same level as at baseline (Fig 2).

**Fig 3:**
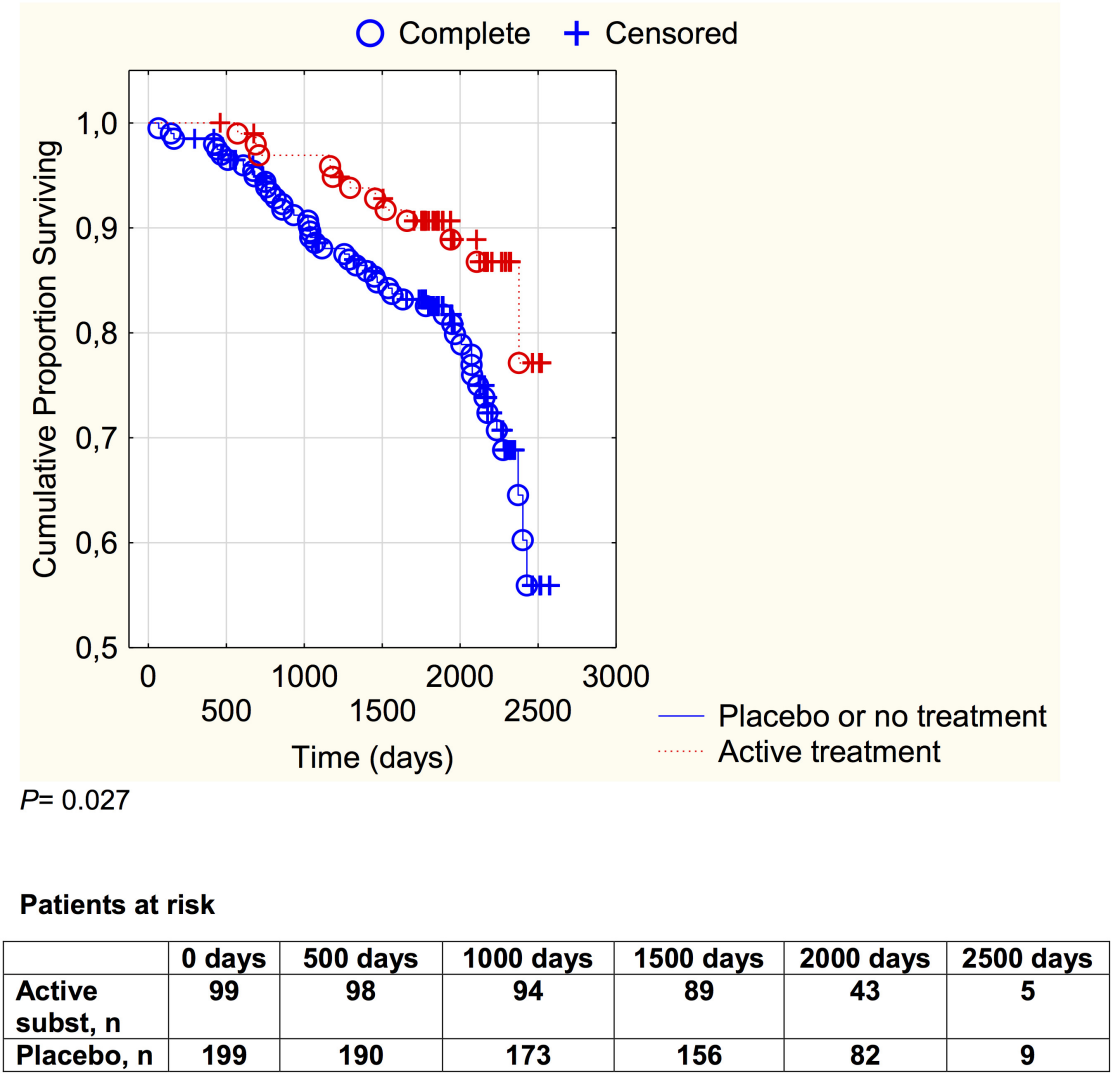
Kaplan-Meier graph illustrating cardiovascular mortality in the study population with a plasma selenium concentration <65 μg/L divided into those given selenium and coenzyme Q10 combined versus those given placebo or no treatment during a follow-up period of 5.2 years. Censored: patients who were still alive at the end of the follow-up period, or who died from non-cardiac causes. Complete: Patients who died from cardiovascular causes during the follow-up period.

In the group with a serum selenium concentration of <65 μg/L, a cardiovascular mortality in the combined control group of 24.1% (48 out of 199) was noted, whereas in the active intervention group a cardiovascular mortality of 12.1% (12 out of 99) was noted. The difference in cardiovascular mortality as a result of the intervention was statistically significant (χ^2^:5.92; *P* = 0.015) and an absolute risk reduction of 12.0% (95%CI 3.24–20.75) could be demonstrated. In order to save one patient, 8.3 patients need to be treated. The cardiovascular mortality during the follow-up period is illustrated in a Kaplan-Meier graph of this group in Fig 3.

The risk reduction observed as a result of the intervention in the lower group was evaluated by a multivariate Cox proportional hazard regression analysis, as shown in Table 2.

**Table 2:** Proportional hazard regression analysis of cardiovascular mortality in the study population with a basal selenium level < 65 μg/L (mean value) during an intervention time of four years.

Variable	Hazard ratio	95% CI	*P*-value
Male	1.17	0.67–2.04	0.57
Smoking	1.84	0.96–3.52	0.07
IHD	1.02	0.59–1.77	0.94
EF<40%	2.23	1.15–4.30	0.02
Active treatment	0.50	0.26–0.95	0.03

Notes: CI: Confidence interval; COPD; EF: Ejection fraction. EF as obtained by echocardiography; IHD: Ischemic heart disease.

From this it could be seen that the intervention resulted in a substantial reduction in risk of cardiovascular mortality (HR: 0.50; 95%CI 0.26–0.95; *P* = 0.03), even when competing covariates were taken into account.

**Fig 4:**
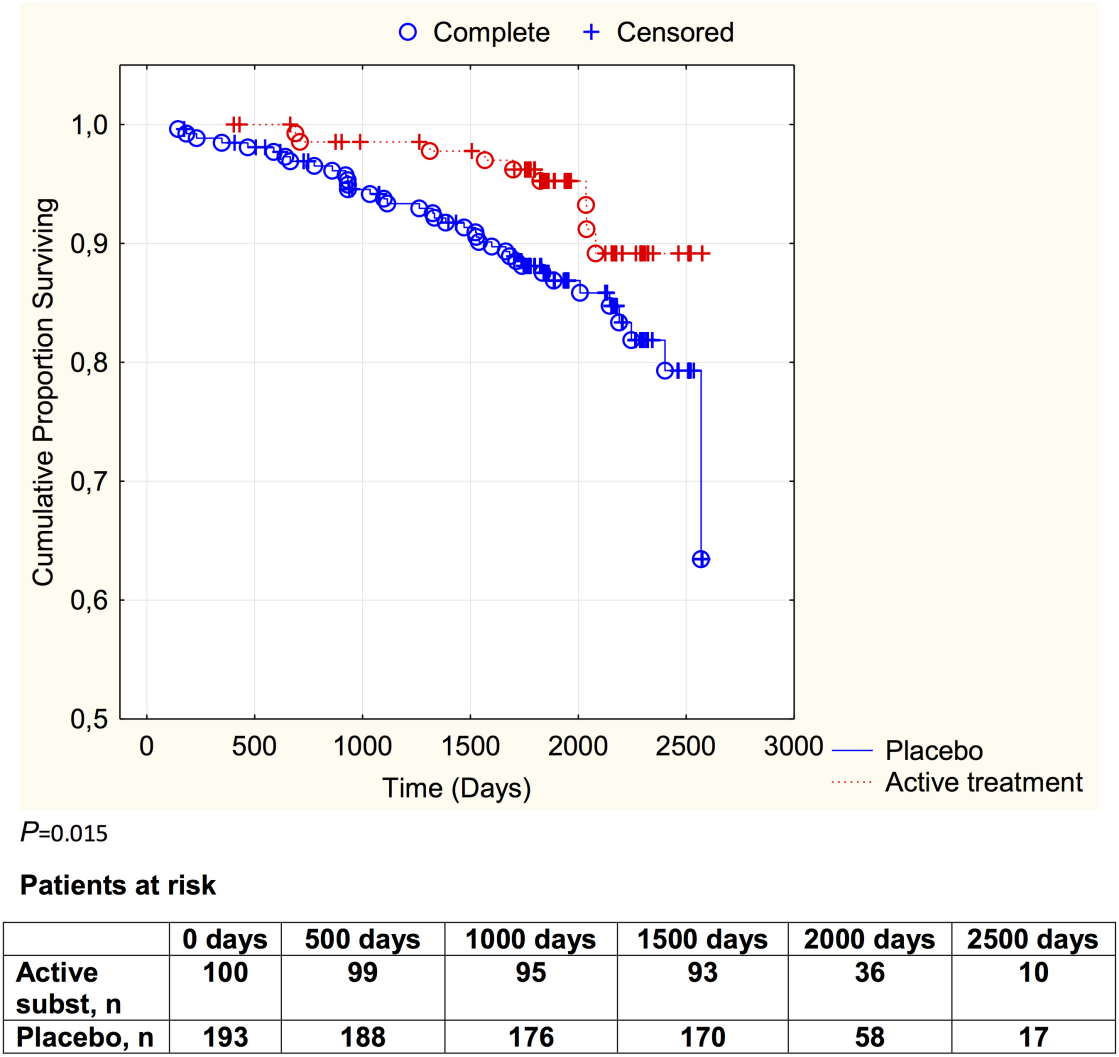
Kaplan-Meier graph illustrating cardiovascular mortality in the study population with a plasma selenium concentration of 65–85 μg/L divided into those given selenium and coenzyme Q10 combined versus those given placebo or no treatment during a follow-up period of 5.2 years. Censored: patients who were still alive at the end of the follow-up period, or who died from non-cardiac causes.

Evaluating the group with a serum selenium concentration between 65 μg/L to 85 μg/L a cardiovascular mortality in the combined control group of 14.0% (27 out of 193) could be seen, as compared with 6.0% (six out of 100) in the active treatment group, thus there was a significantly reduced cardiovascular mortality in the active treatment group (χ^2^:4.21; *P* = 0.040). An absolute risk reduction of 8.0% (95%CI 1.24–14.74) was found, and in order to save one patient 12.5 patients need to be treated. The cardiovascular mortality during the follow-up period is illustrated in a Kaplan-Meier graph of this group in Fig 4.

Evaluating the risk reduction by applying selenium and coenzyme Q10 to the middle selenium concentration group demonstrated a highly significant risk reduction (HR: 0.38; 95%CI 0.15–0.92; *P* = 0.03), even in competition with the same covariates as applied above (Table 3).

**Table 3:** Proportional hazard regression analysis of cardiovascular mortality in the study population with a basal selenium level of 65–85 μg/L (mean value) during an intervention time of four years.

Variable	Hazard ratio	95% CI	*P*-value
Male	1.04	0.50–2.20	0.91
Smoking	3.19	1.24–8.21	0.02
IHD	2.05	0.96–4.41	0.06
EF<40%	2.84	1.17–6.89	0.02
Active treatment	0.38	0.15–0.92	0.03

Notes: CI: Confidence interval; COPD; EF: Ejection fraction. EF as obtained by echocardiography; IHD: Ischemic heart disease.

Finally, evaluating the group with a serum concentration of selenium of > 85 μg/L, we observed a cardiovascular mortality of 17.5% (10 out of 57) in the combined control group, and 15.0% (three out of 20) in the active treatment group, which was not significantly different (*P* = 0.79).

On evaluating the possible risk reduction when given selenium and coenzyme Q10 in the high selenium group through a univariate Cox proportional hazard regression we found no significant risk reduction (HR: 0.90; 95%CI 0.25–3.27; *P* = 0.87). However, again, the sample size was small and the confidence interval was wide, so it is not possible to draw any conclusions from the observations in this group.

The number of participants having a serum level of selenium >105 ug/L, the level that enables full expression of SEPP1, was low, 11 out of 668 individuals (1.6%), and there were only four deaths, of which none had received active treatment. It was therefore not possible to perform any statistical evaluations on this group. Apparently, the baseline suboptimal selenium status strongly influenced the effect of the intervention also in this relatively narrow concentration span.

## Discussion

The present study has shown that those with a low selenium concentration (<65 μg/L) have a significantly higher cardiovascular mortality compared to those with high selenium concentration (>85 μg/L), which is in accordance with the literature [16, 29]. This is also in agreement with the EVA study (Etude du Vieillissement Artériel) that reported an association between low plasma concentration of selenium and mortality [30].

That the supplementation had an effect on the selenium concentration could be seen in Fig 1, and it could also be demonstrated that the fixed selenium dose used within the selenium concentration range seen in the study population gave approximately the same selenium concentration independent of the basal selenium concentration after the given dose.

Furthermore, in the group with the low selenium concentration (<65 μg/L), the supplementation with selenium and coenzyme Q10 resulted in a substantial reduction in the risk of cardiovascular mortality during the observation period. The reduction in cardiovascular mortality was also substantial in the group having a selenium concentration of 65–85 μg/L. However, in the group having a concentration of >85 μg/L the minor mortality reduction did not attain statistical significance and it was not possible to determine whether there was a true reduction or not. First, the sample size of this group was limited, as only 14.5% of the total study population had a selenium concentration >85 μg/L. By dividing the group into subjects receiving active treatment, those receiving placebos, and untreated controls, the sample size was reduced even more and finally, the number of deaths in this group was too small to perform statistical evaluations; 10 in the control group and only three in the active treatment group.

However, by evaluating the first and fourth quartiles of basal selenium concentration, a substantial difference in effect of the supplementation could be demonstrated, indicating there is a greater effect if a more pronounced selenium deficiency exists (19).

The result could also indicate that if a lesser need for supplementation exists, no positive result should be expected, as indicated from the French SU.VI.MAX (SUpplementation en VItamines et Minéraux AntioXydants) study [31]. This study, which did not find any effect of supplementation of antioxidants and selenium on cardiovascular mortality, reported an average selenium concentration in plasma at baseline of 1.09 μmol/L (86 μg/L) in women and 1.14 μmol/L (90 μg/L) in men. The findings of that study are compatible with our findings of small or no effects in those with a higher basal selenium level, which might be explained by the existence of an already sufficient selenium supply and an optimized level of SEPP1 and other selenium enzymes. [31].

The supplementation used in our study clearly raised the selenium concentration (Fig 2), and it could also be seen that the fixed selenium dose resulted in approximately the same selenium concentration in our supplemented subgroups after four years. The serum selenium concentrations achieved after supplementation would secure full expression of the selenoproteins.

Reports from the Nordic countries indicate that the selenium intake is low there [32–34]. However, Finland is an example of the opposite, since the authorities in 1986 decided to carry out selenium supplementation through fertilizers [35]. This increased the selenium intake in Finland significantly, compared to the other Nordic countries [36].

A low selenium intake results in a selenium concentration below the levels for optimum expression of both GPX and SEPP1. Our Nordic group has recently reported suboptimal plasma concentrations of selenium (mean value 67 μg/L) in an elderly “healthy” municipality population in the south of Sweden [19]. Based on the data reported by Xia and co-workers regarding the interrelationship between selenium and coenzyme Q10 (ubiquinone) [37], an intervention with both substances for a four-year-period was performed in the main study of our intervention project. The main results have been published recently [20], showing a significant reduction in cardiovascular mortality in the supplemented group. However, the study did not evaluate whether the effect of the combined intervention differed depending on the plasma selenium concentration at inclusion. The present post-hoc analysis of the aforementioned study has therefore stratified the investigated population into three groups, depending on serum selenium concentration at inclusion. Positive effects of the combined supplementation were clearly demonstrated in those with a suboptimal selenium level at inclusion. More effective results of the supplementation could be observed in those with lower levels of serum selenium at inclusion. Furthermore, we were able to report that in those with selenium levels approximately in the range needed for optimal function of SEPP1, no obvious effects of the supplementation could be observed. Finally, it should be emphasized that in our examined study population of elderly Swedes, a clear need for higher selenium intakes existed, since the basic level was low and only a small proportion, 1.6%, had adequate levels for optimizing the expression of SEPP1 and other selenoproteins.

## Limitations

Some limitations of our study could be identified. The most important one was the limited size of the subgroups, generally resulting in loss of statistical power and uncertainties regarding the obtained results, as well as making an even more detailed subgroup division impossible. Secondly, the study population was an elderly healthy population in Sweden with generally low selenium concentrations, and few participants had higher selenium concentrations sufficient to optimize expression of selenoproteins. This limits the possibilities for comparison of the effect of supplementation between the groups with low selenium and the group with high selenium in our study population.

Also, the included population consisted of persons in a relatively narrow age span, so care should be taken in extrapolating the results to other age strata.

Finally, the included population consisted of a homogenous Caucasian population, and extrapolation of the present observations to other populations may be limited.

However, in spite of the above presented limitations, we believe that the information provided is interesting and important.

## Conclusion

In an elderly healthy Swedish population, higher cardiovascular mortality could be reported among those with a serum selenium concentration <65 μg/L in comparison with those having a concentration of >85 μg/L during a follow-up period of 5.2 years.

The effect of dietary supplementation over four years with selenium and coenzyme Q10 combined in three groups (<65 μg/L, 65–85 μg/L and >85 μg/L) was evaluated. In the two lower groups, substantial reductions of cardiovascular mortality were demonstrated, whereas in the group with the highest selenium concentration, no significant reduction of mortality could be demonstrated. Possibly, no need for supplementation existed in order to obtain full expression of one of the important selenoproteins—selenoprotein P. The results are important and indicate that supplementation with selenium and coenzyme Q10 could be considered in the vast majority of populations of areas where the soil has low selenium content.

## Supporting Information

S1 Protocol Study Protocol. (DOCX)
