# Supplementary material for: Supplementation with Selenium and Coenzyme Q10 Reduces Cardiovascular Mortality in Elderly with Low Selenium Status. A Secondary Analysis of a Randomised Clinical Trial
Source: PLoS One. 2016 Jul 1;11(7):e0157541. doi: 10.1371/journal.pone.0157541 (PMC4930181; doi:10.1371/journal.pone.0157541)
Supplement: S1 Protocol — (DOCX) [file pone.0157541.s001.docx]

**Kisel-10- a prospective randomized double-blind placebo controlled study of the results of intervention with selenium and coenzyme Q10 combined, regarding effect with emphasis on cardiovascular mortality and morbidity.**

**Background**

The amount of patients suffering from heart failure is increasing throughout the Western hemisphere. The prognosis of heart failure is poor, in many cases worse than malignant diseases.

Several studies have demonstrated that by evaluating the plasma concentration of the natriuretic peptides that are stimulated by increased wall tension of the myocardium, it is possible to estimate the risk of cardiovascular mortality.

Scientific reports have demonstrated decreased symptoms of heart failure after selenium supplementation. Selenium, which is acquired by the body through food, often exists in low levels in the body in the northern parts of Europe due to low levels of selenium in the soil. Animal studies have shown that selenium has a positive effect on the myocardium.

Ubiquinon, commonly called Q10, has a central position in many of the enzyme systems of the body. It has recently been shown that the body requires the presence of Q10 to produce selenium containing enzymes which are central in the energy handling processes of cells.

During the study all participants will be followed-up with blood samples, a new patient record, and new tablets every six months. Evaluation of health related quality of life will be performed at inclusion, after 18 months and after 48 months. The quality of life will be evaluated using the validated instrument SF-36. At the end of the study, new blood samples and new echocardiograms will be obtained.

**Aim**

To evaluate changes in primarily cardiovascular mortality and all-cause mortality, and secondary, cardiac function as evaluated with cardiac natriuretic peptides and echocardiography after dietary supplementation of selenium and Q10, or a placebo, in an elderly population during 48 months of intervention.

**Secondary objectives**

To evaluate the cost-effectiveness of selenium and Q10 combined during an intervention of 48 months. To measure the perceived health related quality of life during intervention.

Possible health related economic consequences will be evaluated using the instrument EQ-5D.

**Endpoint**

Development of cardiovascular disease, worsening of existing cardiovascular disease, and cardiovascular mortality during intervention with selenium and Q10 in comparison with placebo.

Change of concentration of natriuretic peptides during intervention with selenium and Q10.

**Secondary endpoint**

Development of all-cause mortality during intervention with selenium and Q10.

Consumption of hospital resources during intervention with selenium and Q10 in relation to placebo.

To evaluate possible mechanisms of interventions as seen in blood samples if effects could be demonstrated as described in the primary endpoint.

**Study design**

A prospective randomized double-blind placebo controlled study.

**Randomizing**

The randomising procedure will be performed using PharmaNord’s computer assisted randomization in blocks of 6.

**Study**

All participants will be offered a selenium-containing yeast preparation corresponding to 200 micrograms of selenium/day, and ubiquinone corresponding to 200 milligrams/day, or a placebo.

During the intervention period, blood samples will be drawn every six months from all participants, and the following list of substances are to be analyzed:

1. NT-proBNP
2. Thioredoxin
3. Thioredoxinreductase
4. Lipidperoxidase
5. Panel of cytokines
6. “New” biomarkers for heart failure
7. Panels of biomarkers for inflammation, oxidative stress, and ischemia.

All blood sample results will be blinded for both the investigators and the participants.

**Inclusion criteria**

Individuals living in the municipality of Kinda, aged between 70-80 years who have accepted participation in the study, and who are expected to fulfill a study period of 4 years.

**Exclusion criteria**

Recent myocardial infarction (within 4 weeks).

Planned cardio-vascular operative procedure within 4 weeks.

Hesitation concerning if the candidate can decide for him/herself whether to participate in the study or not, or if he/she understands the consequences of participation.

Serious disease that substantially reduces survival or where it is not expected that the participant can cooperate for the full 4 year period.

Other factors making participation unreasonable, such as long/complicated transport to the Primary Health Center where the project is managed, or drug/alcohol abuse.

**Preparations**

Coenzyme Q10 ( Bio-Quinon 200mg, PharmaNord, Vejle, Denmark)

Selenium (SelenoPrecise 200 microgram, PharmaNord, Vejle, Denmark)

Placebo (PharmaNord, Vejle, Denmark)

All preparations will be ingested twice a day together with food.

The preparations will be supplied by PharmaNord, Vejle, Denmark. The preparations will be stored in a locked, dry, dark room where only the personnel involved in the study will have access. A rigorous log regarding received and delivered preparations will be kept.

**Concomitant pharmacological treatment**

Participants who have been prescribed pharmacological drugs due to concomitant diseases/medical conditions are advised to continue treatment.

Participants who have been prescribed anticoagulants containing warfarin are advised to continue treatment, but it is recommended that they analyze the INR at the start of the intervention with selenium and Q10, and again after 2 weeks. Interactions between selenium/Q10 and warfarin have been discussed in literature but have not been demonstrated.

**Where is the code list?**

The code list containing information of active treatment/placebo is kept in a sealed envelope at PharmaNord, Vejle, Denmark, and the codes will not be broken before the end of the study, or in the case of an emergency where information regarding the intervention is required.

**Side effects**

Serious side effects should be reported to both the National Board of Health and Welfare and PharmaNord.

**Dropouts**

Participants choosing to discontinue the study for any reason will be followed according to the intention to treat principle. All dropouts will be registered, as well as their reason for discontinuing participation, if the participant chooses to supply this information.

Participants who take supplements of selenium or Q10 for any reason outside the study will be regarded as dropouts.

**Ethical permission**

Ethical permission shall be accepted from the Ethical Review Board before the start of the study. Oral and written “patient” information will be given to all participants, as well as a signed copy of the consent form .

**Permission from the Swedish Medical Products Agency**

Contact has been taken with the Medical Products Agency in Sweden regarding permission to use the preparations during the intervention. However, the principal investigator of the project has been orally informed by the Agency that since this was not regarded as a trial of a medication, but rather of food supplement commodities that are readily available as commercial goods, the Agency would not review the study protocol.

**Participants**

500-600 healthy individuals as well as patients treated for various diseases and already participating in the former epidemiological heart failure study in the Kinda municipality.

**Basic power calculation**

Using the assumption that the incidence of occurrences in the placebo group during a follow up of 4 years is 40%, and 28% in the intervention group, i.e. a difference in incidence between the groups of 30% and an absolute difference of 12%, 244 individuals are needed in order to obtain a statistical power of 80% with a significance level of 5%. A dropout frequency of 15% is to be expected, which is why a minimum of 560 individuals is needed in order to obtain significant differences.

**Principal investigator**

Urban Alehagen, Resident, Dept of Cardiology, University Hospital of Linköping

**Co-investigators**

Ulf Dahlström, Professor, Dept of Cardiology, University Hospital of Linköping

Anders Rosén, Professor, Dept of Cellular Biology, University of Linköping

Mikael Björnstedt, Professor, Dept of Pathology, Karolinska University Hospital, Stockholm

*Study protocol in Swedish*

**KiSel-10- en prospektiv randomiserad dubbel-blind placebo kontrollerad studie av intervention med kombinerad behandling med selen och coenzyme Q10 avseende effekt med tonvikt på dödlighet och sjuklighet i hjärtkärlsjukdomar.**

**Bakgrund**

Antalet patienter som drabbas av hjärtsvikt ökar i hela västvärlden. Prognosen för hjärtsvikt är dålig, sämre än för många tumörsjukdomar.

I flera studier har man kunnat visa att man genom blodprovstagning kunnat analysera koncentrationen av sk. Natriuretiska peptider, vilka utsöndras i ökad omfattning vid ökad belastning av hjärtat, och att denna nivå även korrelerar till risk för död i hjärt-kärlsjukdom.

I några vetenskapliga studier har man visat att symptomen av hjärtsvikt minskar vid tillförsel av ämnet selen. Detta ämne som vanligen tillförs via födan, finns i låga nivåer i framförallt norra Europa. Djurstudier har visat att man har en skyddande effekt av selen på hjärtmuskulaturen. Ubiquinon, vanligen kallat Q10, har en central position i många av kroppens enzymsystem. Studier visat att patienter med hjärtsvikt har en lägre nivå av Q10 både i hjärtmuskel liksom i kroppen. Nyligen har man visat att kroppen kräver närvaro av Q10 för att kunna bygga selenhaltiga enzymer, vilka är centrala i cellens energihantering.

Frågeställningen i den planerade studien är att utvärdera om kombinationsbehandling med selen och Q10 kan påverka en äldre population framförallt med avseende på hjärtsvikt och hjärt-kärlrelaterad död.

Under 1998 erhöll samtliga personer i åldern 70-80 år skrivna i Kinda kommun erbjudande om deltagande i en epidemiologisk studie med tonvikt på hjärtsvikt. Av 1130 personer boende i kommunen i den aktuella åldern accepterade 871 personer deltagande. Dessa har undersökts med ny anamnes, med rutinmässig kroppsundersökning, ultraljudsundersökning av hjärtat, blodprovstagning, samt livskvalitetsanalys. Dessa personer kommer att erbjudas deltagande i en uppföljande epidemiologisk hjärtsviktsstudie, men kommer även att erbjudas deltagande i den aktuella interventionsstudien där man erhåller kosttillskott med 200mikrogram selen/dag, samt 200mg Q10/dag, eller placebo.

Utifrån tidigare studier har man inte sett allvarligare biverkningar på de doser som denna intervention kommer att innebära. Preparaten säljs för närvarande fritt i handeln som hälsokost.

Under studiens gång kommer samtliga personer som accepterat deltagande och inkluderats i studien att kontrolleras med förnyade blodprover, förnyad anamnes samt nya interventionspreparat var 6:e månad. Analys av hälsorelaterad livskvalitet kommer att ske vid inklusion, efter 18 månader, samt efter 48 månader. Den hälsorelaterade livskvaliteten kommer att analyseras bl.a. med hjälp av instrumentet SF-36. I samband med avslutande av studien kommer förutom nya blodprover, även ultraljudsundersökning av hjärtat att genomföras.

**Syfte**

Att kartlägga förändringar av kardiovaskulär samt totalmortalitet och i andra hand påverkan på hjärtfunktion mätt med natriuretiska peptider samt ekokardiografi vid intervention med kosttillskott i form av selen och Q10, eller placebo, hos en äldre öppenvårdspopulation under 48 månaders behandling.

**Secondary objective**

Att utvärdera cost-effectiveness vid intervention med kombinationen selen och Q10 under 48 månaders behandling. Patiens upplevda hälsorelaterade livskvalitet under interventionen.

Eventuella hälsoekonomiska konsekvenser kommer att analyseras med hjälp av instrumentet EQ5D

**Endpoint**

Utveckling av hjärt-kärlsjukdom, försämring av redan befintlig hjärt-kärlsjukdom, samt hjärt-kärl död vid intervention med selen-Q10 jämfört med placebo

**Secondary endpoints**

Utveckling av all-cause mortalitet under intervention med selen-Q10.

Förändring av koncentration av natriuretiska peptider utveckling under intervention med selen-Q10

Slutenvårdskonsumption vid intervention med selen-Q10 i relation till placebo under interventionstiden.

**Studiedesign**

En prospektiv randomiserad dubbel-blind placebo kontrollerad studie

**Randomisering**

Randomisering kommer att ske genom PharmaNords försorg genom dator simulerad randomisering i block om 6.

**Studieupplägg**

Samtliga deltagande personer kommer att erbjudas A. selenjästpreparat motsvarande selen 200 mikrogram/dygn, samt ubiquinon 200 mg/dygn, eller B. Placebo

Under interventionstiden kommer var 6:e månad blodprovstagning att genomföras på deltagarna där följande paneler är planerade att analyseras:

1. NT-proBNP
2. Thioredoxin
3. Thioredoxinreduktas
4. Lipidperoxidas
5. Cytokinpanel
6. ”Nya” biomarkörer för hjärtsvikt

Samtliga provresultat kommer att vara blindade för både prövare samt deltagare

**Inklusionskriterier**

Personer skrivna i Kinda kommun i åldern 70-80år som är accepterat deltagande i interventionsstudien, och som förväntas kunna klara medverkan under 4år.

**Exklusionskriterier**

Nylig genomgången hjärtinfarkt, dvs inom 4 veckor,

Planerad hjärt-kärloperation inom 4 veckor

Tveksamhet att kunna själv fatta beslut om deltagande, eller konsekvensen av detta.

Allvarlig sjukdom som begränsar överlevnaden, och där man inte förväntar att deltagaren kan medverka under 4 år.

Andra faktorer som gör att medverkan inte förefaller rimlig, tex. Lång/komplicerad transport till Vårdcentralen, överförbrukning av t.ex. alkohol.

**Preparat**

Coenzym Q10 (Bio-Quinon 200mg, PharmaNord, Vejle, Danmark)

Selen (SelenoPrecise 200 mikrogram, PharmaNord, Vejle, Danmark)

Eller Placebo (PharmaNord, Vejle, Danmark)

Preparaten kommer att erhållas ifrån PharmaNord, Vejle, Danmark. Preparaten kommer att förvaras i låst, torrt mörkt utrymme, där endast forskningsrelaterad personal har nyckel. Noggrann log kommer att föras över erhållna samt utlämnade preparat.

**Intag av preparaten**

Intag av projektpreparaten skall ske två gånger dagligen samt tillsammans med föda.

**Farmakologisk behandling**

Deltagare i studien som insatts på farmakologisk behandling mot sjukdom/medicinskt tillstånd skall fortsätta med ordinerad behandling. Deltagare som ordinerats antikoagulansbehandling med warfarin skall fortsätta med denna, men rekommenderas genomföra extra INR-kontroll i samband med interventionsstart med selen och Q10, samt 2 veckor efter start. Interaktion mellan Q10 och warfarin har diskuterats i litteraturen, men ej kunnat påvisas.

**Var finns kodlistan för projektet?**

Kodlistan kommer att förvaras i förslutet kuvert hos PharmaNord, och koderna kommer inte brytas innan deadline för projektet, eller vid akuta tillstånd som kräver information om studiepreparaten.

**Biverkningar**

I händelse av allvarlig biverkan skall denna rapporteras både till Socialstyrelsen, liksom till fabrikanten.

**Avhopp**

Deltagare som av en eller annan orsak väljer att avbryta deltagande kommer att följas enligt principen intention to treat principen. Alla avhopp kommer att registreras liksom orsak till detta, om deltagaren är villig att delge detta.

Deltagare som av någon anledning tar kompletterande supplement med selen eller Q10 utanför studiens ram under pågående projekt kommer att betraktas som avhopp.

**Etiktillstånd**

Skall sökas och erhållas innan studiestart. Muntlig och skriftlig information skall ges till varje deltagare, och en signerad kopia av det skriftliga medgivandet skall distribueras till varje deltagare.

**Godkännande ifrån Läkemedelsverket**

Kontakt har tagits med Läkemedelsverket för att utröna om tillstånd krävs för at kunna driva studien. Ansvarig för projektet har muntligen blivit informerad om att eftersom de ingående preparaten som kommer att användas vid intervention försäljs fritt i livsmedelshandeln omfattas inte dessa preparat av Läkemedelsverkets kontroll, och kommer därför inte att bedömas av Läkemedelsverket.

**Försöksobjekt:**

500-600 friska samt patienter deltagande i det tidigare epidemiologiska hjärtsviktsprojektet i Kinda kommun

**Grundläggande powerberäkning:**

Vid antagande att incidensen av inträffade händelser i kontrollgruppen under en uppföljningstid av 4 år är 40%, och i interventionsgruppen är 28%, dvs en procentuell skillnad mellan grupperna i incidens på 30%, och en reell skillnad på 12% så krävs för att erhålla en statistisk styrka (power) på 80% och på en 5% signifikansnivå att 244 individer inkluderas i vardera gruppen, I dylika studier brukar man även kalkylera för ett bortfall i storleksordningen 15%, vilket innebär att drygt 560 individer måste inkluderas i studien för at uppnå skillnader.

**Ansvarig för projektet:**

Urban Alehagen Specialistläkare, Kard klin, US

**Medansvariga:**

Ulf Dahlström, Professor, Kard klin, US

Anders Rosen, Professor, Avd för Cellbiologi, US

Mikael Björnstedt, Professor, Avd för Patologi, Karolinska Sjukhuset, Stockholm
